# Precision medicine and mitral valve assessment

**DOI:** 10.1093/ehjimp/qyaf073

**Published:** 2025-06-02

**Authors:** Edgar Argulian, Julia Grapsa

**Affiliations:** Department of Cardiology, Mount Sinai Morningside, Icahn School of Medicine at Mount Sinai, 1111 Amsterdam Avenue, New York, NY 10025, USA; Department of Cardiology, Brigham and Women’s Hospital, Harvard Medical School, Boston, MA 02115, USA

Accurate grading of mitral regurgitation severity is essential in optimal patient management. Nowadays that more patients have a complex anatomy, and they live longer, the imager is required to provide a very comprehensive assessment of complex mitral jets.

## Mitral regurgitation assessment made easy

**Figure 1:**
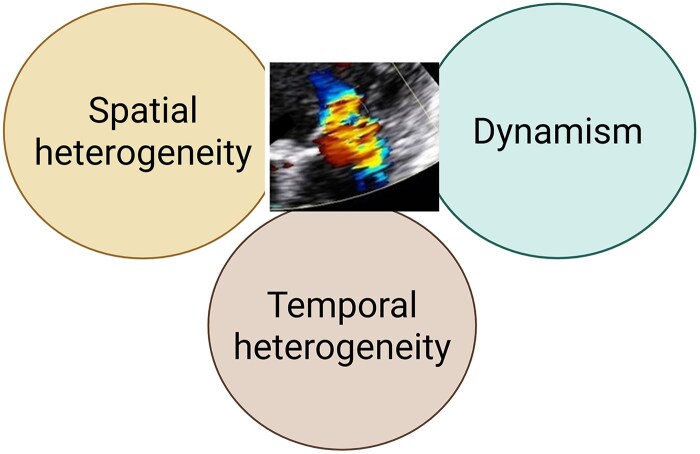
Domains necessary for mitral regurgitation grading.

Complex and elaborate schemes largely based on echocardiographic assessment have been proposed, which are equally applicable to different mitral regurgitation mechanisms. Fundamentally, these schemes integrate three basic categories: measures describing the lesion severity; measures describing left ventricular adverse remodelling; and measures of haemodynamic impact.^1^ However, different mechanisms of mitral regurgitation have different pathophysiologic milieu requiring tailored approaches to severity grading and to thresholds for therapeutic interventions. The following domains should be adopted in mitral regurgitation grading, given that the most commonly used measures of severity grading rely on jet characteristics: spatial heterogeneity, temporal variation, and dynamism (*Figure 1*). Spatial heterogeneity refers to the complexity and variation of the vena contracta shape. Temporal heterogeneity refers to systolic variation in mitral regurgitation flow rate. Dynamism describes changes in mitral regurgitation severity depending on loading conditions, daily activities, and treatment regimen.

Chronic primary mitral regurgitation due to mitral leaflet and chordal abnormality is largely a volume overload problem. Excess volume generated by the mitral valve pathology leads to adverse left ventricular remodelling and eventually haemodynamic failure resulting in elevated filling pressures and pulmonary hypertension. Spatial heterogeneity is reflected by various orifice shapes, especially with eccentric jets, making conventional quantification difficult. Temporal heterogeneity is common with mitral valve prolapse which typically accentuates in mid and late systole. Failure to account for temporal variation can lead to overestimation of the regurgitation severity. Dynamism can be present with primary regurgitation of different aetiologies, but it is usually not as substantial as with secondary mitral regurgitation. For chronic primary mitral regurgitation, precise volumetric assessment is paramount, as volume overload represents the initial and most critical pathophysiologic driver of disease progression. Echocardiographic evaluation, particularly when reliant on jet characteristics, has recognized limitations, even in patients with distinct valvular pathology such as a flail leaflet or specific echocardiographic phenomena like the Coanda effect.^2^ In contrast, cardiac magnetic resonance imaging enables accurate and prognostically meaningful quantification of mitral regurgitation severity, making it a valuable tool in borderline or equivocal cases.^3^ Notably, one study demonstrated a strong correlation between preoperative regurgitant volume measured by cardiac magnetic resonance imaging and the degree of left ventricular reverse remodelling following surgical intervention.^4^

## The role of haemodynamics

Secondary ventricular mitral regurgitation appears to be largely a haemodynamic problem. It has now been divided into two major phenotypes: ventricular and atrial. Ventricular remodelling leads to secondary changes in the subvalvular apparatus and mitral valve configuration resulting in a coaptation gap. Spatial heterogeneity is common, resulting in oblong and elliptical vena contracta in symmetric leaflet tethering and elongated and asymmetric vena contracta in ischaemic mitral regurgitation due to tethering of the medial portion of the posterior leaflet. Temporal heterogeneity is ubiquitous, usually caused by mid-systolic drop in regurgitation flow rate due to interaction of the closing and tethering forces. Dynamism is the most important feature of secondary mitral regurgitation: the regurgitation severity can change dramatically depending on loading conditions, daily activities, and treatment regimen. Regurgitation severity captured on resting echocardiogram may not reflect the true haemodynamic load of mitral regurgitation experienced by the patient. More importantly, the crucial measure of mitral regurgitation impact, both symptomatic and prognostic, is not necessarily the snapshot regurgitant volume, but its effect on the left atrial pressure. This has been demonstrated in several haemodynamic studies in patients undergoing mitral edge-to-edge repair: both the degree of residual mitral regurgitation and post-procedural left atrial pressure appear to be the major determinants of post-procedure prognosis.^5^ Similarly, in echocardiographic studies, improvement in pulmonary venous systolic flow captured by pulsed-wave Doppler is a strong predictor of post-procedure events.^6^ Interestingly, in contemporary studies, substantial improvement in echocardiographic mitral regurgitation grade is achieved in vast majority of patients undergoing edge-to-edge repair, but this does not necessarily translate into uniform and substantial decrease in left atrial pressure.

With regards to atrial mitral regurgitation, it is most common in heart failure with preserved ejection fraction and/or atrial fibrillation. Depending on mitral pathology, regurgitation can be subdivided into central or eccentric creating differences in left ventricular and atrial remodelling.^7^ Malcoaptation of the leaflets creates the central jet in the middle of the valve. In a smaller subgroup of patients (20–30%), eccentric MR jets are located posterior due to ‘hamstringing of the posterior MV leaflet’.^7^ Often, atrial MR is accompanied by overriding of the anterior leaflet due to excessive annular dilation and a form of tethering known as *atriogenic tethering*^7^ because the subvalvular apparatus and the left ventricle remain completely intact. Eccentric jets may usually need cross-sectional imaging for accurate quantification such as transesophageal echocardiography.

LV remodelling became a hot topic of discussion after major TEER trials and the terms proportionate and disproportionate MR is an important contribution in the understanding of MR.^8^

In the harmonious relationship of echocardiographic with haemodynamics, it is reasonable to assume that it is not necessarily the exact volumetric grade of the mitral regurgitation but rather its contribution to the increased left atrial pressure that matters in secondary mitral regurgitation. This contribution is determined by the balance of regurgitation severity and dynamism, degree of left ventricular remodelling, left atrial and left ventricular stiffness, and other haemodynamic factors. Therefore, even moderate appearing mitral regurgitation by echocardiographic grade can contribute to haemodynamic worsening and proved to be a therapeutic target. The grading of secondary mitral regurgitation should focus of haemodynamic measures of regurgitation impact rather than absolute volumetric quantification. This can be achieved by both echocardiographic and invasive haemodynamic measures of increased pressures at rest and comprehensive assessment with low load exercise to uncover mitral regurgitation dynamism.

Certain mechanisms of mitral regurgitation fall beyond the traditional categories and require further understanding. Atrial functional mitral regurgitation due to annular dilation and occasionally substantial hamstringing of the posterior leaflet represent a unique challenge. The therapeutic approach to this entity is evolving. Similarly, truly mixed mitral pathology does exist in some patients. Interestingly, in pathologic studies of patients with secondary mitral regurgitation some degree of mitral leaflet degeneration has been demonstrated questioning the notion of ‘normal leaflets’ in these patients.

**Figure 2:**
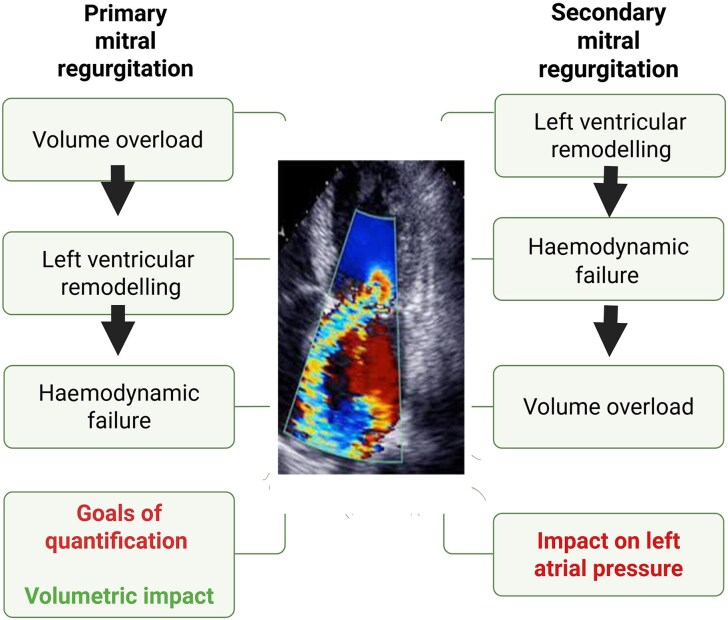
Differences in primary and secondary mitral regurgitation regarding pathophysiology and regurgitation quantification goals.

Primary and secondary mitral regurgitation share a common designation but represent fundamentally distinct pathophysiologic entities, a distinction that has long been recognized. Consequently, the approach to grading mitral regurgitation severity should be tailored to the underlying mechanism of the disease (*Figure 2*).

## Future is bright

As we move along to a second revolution of mitral valve devices, the association of haemodynamics, appropriate and personalized mitral assessment in conjunction of left ventricular and atrial evaluation will be key in the optimal selection of patients and reduction of post-procedural complications.

## Data Availability

Not applicable.
